# Population Pharmacokinetics of Busulfan and Its Metabolite Sulfolane in Patients with Myelofibrosis Undergoing Hematopoietic Stem Cell Transplantation

**DOI:** 10.3390/pharmaceutics14061145

**Published:** 2022-05-27

**Authors:** Adrin Dadkhah, Sebastian Georg Wicha, Nicolaus Kröger, Alexander Müller, Christoph Pfaffendorf, Maria Riedner, Anita Badbaran, Boris Fehse, Claudia Langebrake

**Affiliations:** 1 Hospital Pharmacy, University Medical Center Hamburg-Eppendorf, 20251 Hamburg, Germany; clangebrake@uke.de; 2 Department of Clinical Pharmacy, Institute of Pharmacy, University of Hamburg, 20146 Hamburg, Germany; sebastian.wicha@uni-hamburg.de (S.G.W.); christoph.pfaffendorf@uni-hamburg.de (C.P.); 3 Department of Stem Cell Transplantation, University Medical Center Hamburg-Eppendorf, 20251 Hamburg, Germany; n.kroeger@uke.de (N.K.); badbaran@uke.de (A.B.); fehse@uke.de (B.F.); 4 Department of Legal Medicine, University Medical Center Hamburg-Eppendorf, 20251 Hamburg, Germany; alexander.mueller@uke.de; 5 Technology Platform Mass Spectrometry, University of Hamburg, 20146 Hamburg, Germany; maria.riedner@uni-hamburg.de

**Keywords:** busulfan, sulfolane, myelofibrosis, population pharmacokinetics

## Abstract

For patients with myelofibrosis, allogeneic hematopoietic stem cell transplantation (allo-HSCT) remains the only curative treatment to date. Busulfan-based conditioning regimens are commonly used, although high inter-individual variability (IIV) in busulfan drug exposure makes individual dose selection challenging. Since data regarding the IIV in patients with myelofibrosis are sparse, this study aimed to develop a population pharmacokinetic (PopPK) model of busulfan and its metabolite sulfolane in patients with myelofibrosis. The influence of patient-specific covariates on the pharmacokinetics of drug and metabolite was assessed using non-linear mixed effects modeling in NONMEM^®^. We obtained 523 plasma concentrations of busulfan and its metabolite sulfolane from 37 patients with myelofibrosis. The final model showed a population clearance (CL) and volume of distribution (V_d_) of 0.217 L/h/kg and 0.82 L/kg for busulfan and 0.021 L/h/kg and 0.65 L/kg for its metabolite. Total body weight (TBW) and a single-nucleotide polymorphism of glutathione-S-transferase A1 (GSTA1 SNP) displayed a significant impact on volume of distribution and metabolite clearance, respectively. This is the first PopPK-model developed to describe busulfan’s pharmacokinetics in patients with myelofibrosis. Incorporating its metabolite sulfolane into the model not only allowed the characterization of the covariate relationship between GSTA1 and the clearance of the metabolite but also improved the understanding of busulfan’s metabolic pathway.

## 1. Introduction

Myelofibrosis is a chronic myeloproliferative disorder that is characterized by a cytokine-mediated fibrosis of the bone marrow. This results in extramedullary hematopoiesis in the liver and spleen, often accompanied by enlargement of both organs [1]. Genetic aberrations of the genes JAK2, MPL and CALR were identified as the cause of this myeloproliferative disorder [2] and deemed relevant to clinical decision-making with regard to prognosis as well [3]. Since comprehensive mutational profiling has shown that most patients carry a JAKV617F mutation, initial therapy with the JAK1/JAK2 inhibitor ruxolitinib may reduce splenomegaly and improve performance status [4]. 

However, allogeneic hematopoietic stem cell transplantation (allo-HSCT) remains the only curative treatment to date. Prior to allo-HSCT, patients typically undergo either reduced intensity conditioning (RIC) [5] or myeloablative conditioning (MAC) [6,7] with busulfan and fludarabine. 

Regarding other neoplastic diseases, the relationship between busulfan drug exposure and patient outcome after allo-HSCT has been investigated extensively. On the one hand, under-exposure is associated with higher risks of relapse and graft rejection, and on the other hand, over-exposure more frequently results in organ toxicity, sinusoidal obstructive syndrome (SOS), acute graft-versus-host disease (aGvHD) and overall higher treatment-related mortality (TRM) [8,9,10,11]. It is also known that busulfan has a high inter-individual variability (IIV) considering the ratio of dose and drug exposure, which makes individual dose selection challenging. Therefore, to maintain the narrow therapeutic range, it is recommended to conduct therapeutic drug monitoring (TDM) for higher dose busulfan in MAC conditioning regimens [9,12].

Studies that have investigated other neoplastic diseases found that patient-specific covariates such as age, weight, body surface area or co-medication might affect the clearance (CL) or volume of distribution (Vd) of busulfan and, therefore, may explain the IIV [13,14,15,16,17,18] as well as the inter-occasion variability (IOV) [14,19,20].

Since patients with myelofibrosis have an elevated risk of hepatotoxicity and impaired liver function due to extramedullary hematopoiesis, PK parameters of busulfan might additionally be affected. However, data describing the pharmacokinetic variability of busulfan prior to allo-HSCT in patients with myelofibrosis are sparse.

Overall, the pharmacokinetics of busulfan are complex, considering that its metabolic pathway is still not fully understood. Lawson et al. describe the conjugation of busulfan with glutathione through different isoenzymes of glutathione-S-transferase (GST), which eventually results in the four major metabolites: tetrahydrothiophene (THT), THT-1-oxide, sulfolane and 3-hydroxysulfolane [21]. Two of the most prominent isoenzymes are GSTA1 and GSTM1, and therefore, their impact on busulfan CL was subject to several investigations. In every population pharmacokinetic (PopPK) model, except one [22], it was found that polymorphisms correlate with a decrease in CL [23,24,25,26]. 

In order to get a better understanding of busulfan’s metabolic pathway, joint PK-modeling of the parent drug and its metabolite seems sensible as it might account for uncertainties in the model and consequently improve the parameter estimations [27]. Moreover, a metabolic ratio of busulfan/sulfolane ≥5 is associated with a higher rate of graft failure and decreased event-free survival (EFS) [28]. However, none of busulfan’s metabolites have yet been incorporated into a PopPK model.

Therefore, this study aimed to develop a PopPK model of busulfan and its metabolite sulfolane by examining known and determining new patient-specific covariates to explain busulfan’s inter-individual variability in patients with myelofibrosis.

## 2. Materials and Methods

### 2.1. Patients and Data Collection

Patients of both sexes aged ≥18 years with diagnosed myelofibrosis that were scheduled for allo-HSCT with previous reduced intensity busulfan/fludarabine conditioning therapy at the University Medical Center Hamburg–Eppendorf between November 2018 and June 2020 were included after written informed consent. We also obtained written consent from patients who already underwent allo-HSCT between October 2016 and October 2017 and met the same criteria for enrollment. The single-center, prospective and partly retrospective, observational study was approved by the local Ethics Committee of the Hamburg Chamber of Physicians on 16 October 2018 (approval number: PV5842) and registered at the German Clinical Trials Register, number DRKS00015217, on 31 October 2018.

Patient demographics and routine clinical data, such as levels of aspartate transaminase (AST), alanine transaminase (ALT), bilirubin, alkaline phosphatase (AP), results of elastography by Fibroscan, and genetic and other diagnostic markers, were obtained from the electronic patient record before busulfan administration. Scores according to the Dynamic International Prognostic Scoring System (DIPSS) for primary myelofibrosis and myelofibrosis secondary to PV and ET (MYSEC) for post-polycythemia vera (Post-PV) and post-essential thrombocythemia (Post-ET) myelofibrosis were determined to predict patient outcomes [29,30,31]. Treatment-related adverse events and outcomes (mucositis, aGvHD, cGvHD, SOS, relapse, death) were evaluated for one year after allo-HSCT. 

Continuous variables are reported as medians with interquartile ranges (IQR) and discrete variables as counts (percentages).

### 2.2. Dosing, Pharmacokinetic Sampling and Quantification

Depending on the therapy standards at the time of enrollment, patients received either 10 doses of i.v. busulfan (0.8 mg/kg) every 6 h with a 2 h infusion rate (Q6H) or were dosed with three busulfan infusions every 24 h with an initial dose of 3.2 mg/kg and a 3 h infusion rate, followed by dose adjustment if necessary to achieve a cumulative area under the curve (cAUC) of 50 mg × h/L (Q24H). Furthermore, all patients received fludarabine and anti-thymocyte globulin (ATG) as part of the conditioning chemotherapy and levetiracetam as anticonvulsant prophylaxis. Comedications that are commonly known for their drug–drug interactions with busulfan, such as phenytoin, metronidazole, or azoles, were not administered during busulfan treatment. 

For Q6H, blood samples were drawn 2.08, 3, 4 and 5.5 h after the start of the first and ninth infusion with an additional trough sample 5.5 h after start of the fifth infusion. For Q24H, sampling was conducted at 3.08, 4, 5 and 6.5 h after the start of the first infusion. Blood samples were drawn into serum tubes, immediately stored at 2–8 °C and centrifuged (2000 rpm, 10 min at 4 °C) shortly after. Supernatant plasma was separated into two aliquots and stored at −80 °C until analysis. 

Busulfan was quantified at the Department of Legal Medicine at the University Medical Center Hamburg–Eppendorf using a validated gas chromatography with mass spectrometric detection method. The quantification of sulfolane was conducted according to the bioanalytical method of McCune et al. using a QTRAP 5500 mass spectrometer (SCIEX, Framingham, MA, USA) coupled with a 1290 Infinity HPLC II (Agilent Technologies, Santa Clara, CA, USA) [32] at the Dept. of Clinical Pharmacy, Institute of Pharmacy, University of Hamburg. A detailed description of the bioanalytical method is provided in the Supplementary Materials.

### 2.3. Genotyping

DNA samples were obtained from bone marrow or peripheral blood samples before transplantation. Genotyping was performed by real-time quantitative polymerase chain reaction (PCR) on a LightCycler 480 II (Roche Diagnostics, Penzberg, Germany). 

In order to find the GSTA1 * B haplotype, which was reported to have a significantly decreased promoter activity [33], we analyzed the DNA for the single-nucleotide polymorphism (SNP) −52G > A (rs3957356) according to the method published by Ansari et al. [24]. GSTM1 deletion was detected as described by Choi et al. [23]. The primer sets used for the genotyping assays are reported in the Supplementary Materials.

### 2.4. PopPK Analysis

PopPK modeling was carried out in NONMEM^®^ (version 7.4.3, ICON, Gaithersburg, MD, USA) using non-linear mixed-effect modeling. First-order conditional estimation with interaction (FOCE-I) between inter-individual and residual random effects was used throughout the process. Pirana version 3.0.1 (Certara, Princeton, NJ, USA) was used as run manager [34] and R version 4.1.2 (R Foundation for Statistical Computing, Vienna, Austria) was used for the exploratory data analysis and graphical postprocessing of the NONMEM^®^ output. 

Nested models were compared using the likelihood-ratio test (alpha = 0.05, one degree of freedom), where a drop in objective function value (OFV) of 3.84 was considered as a significant improvement. Non-nested models were compared by the Akaike information criterion (AIC), for which superior models are indicated by a lower score [35]. Goodness-of-fit (GOF) plots, such as observed vs. population-predicted (PRED) and individual-predicted concentrations (IPRED) or conditional weighted residuals vs. time after dose and PRED, as well as eta shrinkage, were used for evaluation. A shrinkage below 30% was deemed acceptable [36].

Additive and proportional residual error models, as well as a combination of both, were tested to describe residual variability of both busulfan and sulfolane. Since intra-individual variability in busulfan pharmacokinetics is frequently observed during therapy [20], we also tested IOV on both CL and Vd of busulfan and sulfolane. The L2 data item in NONMEM^®^ was used in order to test the correlation between the parent drug and its metabolite concentration measurements.

### 2.5. Covariate Model

After an initial screening for physiological plausibility and subsequent visual inspection to evaluate if there were correlations with individual estimates of the PK parameters, potential covariates were incorporated into the model using linear, exponential or power functions where appropriate. Statistical evaluation was carried out by a stepwise covariate modeling approach with alpha ≤ 0.05 (ΔOFV ≥ −3.84) in the forward inclusion step and alpha ≤ 0.01 (ΔOFV ≥ 6.64) in the backward elimination step. 

Categorical covariates (GSTA1 SNP, GSTM1 deletion, sex, driver mutations) were coded as 0 or 1, whereas continuous variables (age, weight, height, BSA, serum levels, Fibroscan) were centered around their median value. Missing values for Fibroscan were imputed using the population median. 

Eventually, the cAUC of all patients, as well as the clearance of busulfan after the first and ninth dose, were calculated by using the individual estimates of the final model for each patient.

### 2.6. Model Evaluation

Evaluation of the final model was performed by using a prediction-corrected visual predictive check (pcVPC) [37] with 1000 simulations and stratification based on predicted concentrations of the parent and metabolite as well as on the dosing regimen. Subsequently, the sampling-importance resampling (SIR) method (M/m = 5000/1000) was used to evaluate model robustness and determine the 95% confidence intervals (CI) of the estimated parameters [38].

## 3. Results

### 3.1. Patients and Data

In total, 37 patients diagnosed with myelofibrosis undergoing reduced conditioning chemotherapy with busulfan prior to allo-HSCT were included in this study. Thirty patients were included in the prospective part of the study and seven more patients consented to provide their data, measured busulfan plasma concentrations and remaining DNA samples for genotyping for retrospective analysis. The study population consisted of 19 female and 18 male patients, typically aged 60 years (median, IQR 53.5–65.5 years), with a median total body weight (TBW) of 75 kg (IQR 64.05–88.25 kg). Briefly, 18 patients had primary myelofibrosis, whereas 9 and 10 patients were diagnosed with Post-ET or Post-PV myelofibrosis, respectively.

A GSTA1 SNP was found in 28 patients (75.7%), whereas a GSTM1 deletion was detected in 19 patients (51.35%), and 10 patients (27%) had a combination of both. Overall, 523 plasma concentrations of busulfan and sulfolane were included in the PK analysis, from which 282 were parent drug and 241 were metabolite concentrations. In comparison, there were more busulfan plasma concentrations available, since sulfolane plasma concentrations could only be obtained from patients in the prospective part of the study. 

In total, seven plasma concentrations were excluded from the analysis due to mishandling or implausible concentrations, and 70 sulfolane plasma concentrations (13.4% of all plasma concentrations) were below the limit of quantification (BLQ, lower limit of quantification = 0.04 mg/L). The median cAUC of busulfan was 36.06 mg × h/L (range: 25.67–61.85 mg × h/L). Individual busulfan clearance (Figure S1) decreased from 17.16 L/h after the first dose (median, range: 10.55–22.36 L/h) to 16.47 L/h (median, range: 10.05–19.27 L/h) after the ninth dose. An overview of patient characteristics and clinical data is presented in Table 1.

### 3.2. Base Model

The pharmacokinetics of busulfan and its metabolite sulfolane were best described by a one-compartment (1CMT) model with first-order elimination. The addition of a second compartment (2CMT) did not significantly improve the model, as indicated by their AIC (−1956.88 for 1CMT vs. −1956.41 for 2CMT) and lack of improvement in GOF plots. A proportional error model was used to describe the residual variability, since a mixed error model led to high eta shrinkage. The co-variance between the proportional error of busulfan (Prop. σ_Bu_) and sulfolane (Prop. σ_Su_) was implemented by using a sigma block employing the L2 data item. AIC dropped by 66 points when the L2 data item was used, as the concentration between parent drug and its metabolite measurements was correlated (11.8%). The inclusion of IIV on both CL (CL_Bu_ and CL_Su_) and Vd (V_Bu_ and V_Su_) of busulfan and sulfolane considerably improved the model. Before including IOV, each administration, followed by blood sampling, was defined as a new occasion. The implementation of IOV on CL_Bu_ further improved the model (ΔOFV −8.96). BLQ observations were included into the model and accounted for by the error model. 

**Figure 1:**
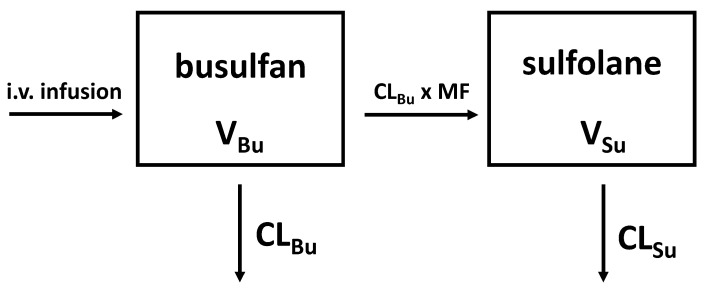
Compartmental model used to describe busulfan and sulfolane plasma concentration–time profiles. CL_Bu_: busulfan clearance, representing the total clearance; V_Bu_: volume of distribution of busulfan; MF: metabolic fraction; CL_Su_: sulfolane clearance; V_Su_: volume of distribution of sulfolane; formation of sulfolane as part of the total clearance.

Overall, busulfan and sulfolane plasma concentration–time profiles were adequately described by the compartmental model presented in Figure 1.

### 3.3. Covariate Model

Initially, 39 demographic or clinical variables were identified as candidates for testing. Graphical exploration revealed 12 of them to be potential covariates, which then were incorporated separately into the model. Lastly, seven covariates (TBW, JAK2 mutation, GSTA1 SNP, GSTM1 deletion, De Ritis ratio, AP and bilirubin) showed a statistically significant drop in OFV (alpha ≤ 0.05) in the forward inclusion step.

Establishing a powered relationship between TBW and V_Bu_ improved the base model statistically from an OFV of −2655.3 to −2686.8 (ΔOFV −31.5), as well as graphically, and reduced the IIV on V_Bu_ from 18.5% to 10.4%. Incorporating JAK2 mutation on CL_Su_ into the model reduced the OFV by 12.6 points to −2699.4; however, parameter estimates then became physiologically implausible, and therefore, it was discarded. The exponential relationship between GSTA1 and CL_Su_ yielded in a drop by 10.7 points (OFV: −2697.5) and reduced the IIV on CL_Su_ from 136.3% to 112.8%. A further reduction by 7.61 points was achieved by including the De Ritis ratio on CL_Bu_. However, this resulted in high relative standard errors of the PK parameters and thus the relationship was not retained in the model. An additional powered relationship between either AP or bilirubin and CL_Bu_ was statistically significant in the forward inclusion step (ΔOFV −5.56 and −5.23) but neither reduced the IIV on CL_Bu_ substantially nor showed notable improvement in GOF plots and, therefore, was eliminated within the backward elimination step.

The final covariate relationship on V_Bu_ (1) and CL_Su_ (2) can be expressed as:

(1) $$V_{\mathrm{Bu}}={\;V}_{\mathrm{Bu}-\mathrm{typ}}\times \left(\frac{\mathrm{TBW}}{75}\;\right){\;}^{0.854}$$

(2) $$\begin{array}{c} {\mathrm{CL}}_{\mathrm{Su}}={\;\mathrm{CL}}_{\mathrm{Su}-\mathrm{typ}}\times {\;e}^{\;1.43}\;\left(\mathrm{for}\;\mathrm{GSTA}1\right)\\ {\mathrm{CL}}_{\mathrm{Su}}={\mathrm{CL}}_{\mathrm{Su}-\mathrm{typ}}\;\left(\mathrm{for}\;\mathrm{non}-\mathrm{GSTA}1\right) \end{array}$$

where V_Bu-typ_ and CL_Su-typ_ are the typical values of V_Bu_ and CL_Su_, 0.854 and 1.43, and describe the effect of TBW and GSTA1 on V_Bu_ and CL_Su_, respectively. 

For the final model, the typical CL_Bu_ and V_Bu_ for a 75 kg patient were 16.3 L/h (IIV: 21.5% CV) and 61.5 L (IIV: 10% CV), respectively. CL and Vd of sulfolane were estimated to be 1.61 L/h (IIV: 112.8% CV) and 48.8 L (IIV: 77.6% CV). IOV on CL_Bu_ was 7.6%. The final model estimates and their 95% CI determined by SIR are provided in Table 2.

### 3.4. Model Evaluation

**Figure 2:**
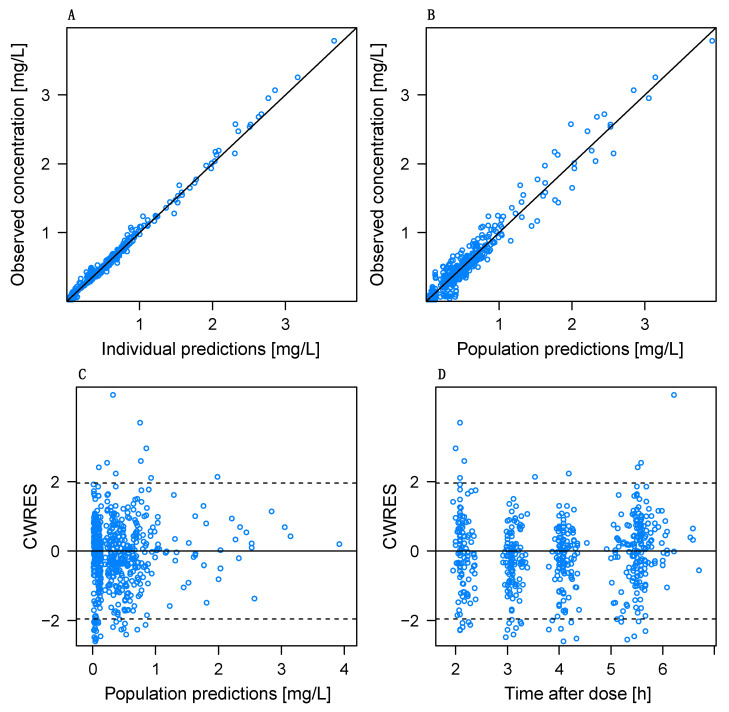
Goodness-of-fit (GOF) plots of the final PK model for busulfan and sulfolane. Observed concentration versus (**A**) individual predicted concentrations, and (**B**) population predicted concentrations; solid line = line of identity. Conditional weighted residuals (CWRES) versus population predicted concentration (**C**) and time after first dose (**D**).

An overview of the GOF plots for the final model is shown in Figure 2. Plots of individual predictions (Figure 2A) as well as population predictions (Figure 2B) against observations depict an even distribution around the identity line. The conditional weighted residuals (CWRES) show a normal distribution around the *x*-axis when plotted against population predictions (Figure 2C) and time after dose (Figure 2D).

**Figure 3:**
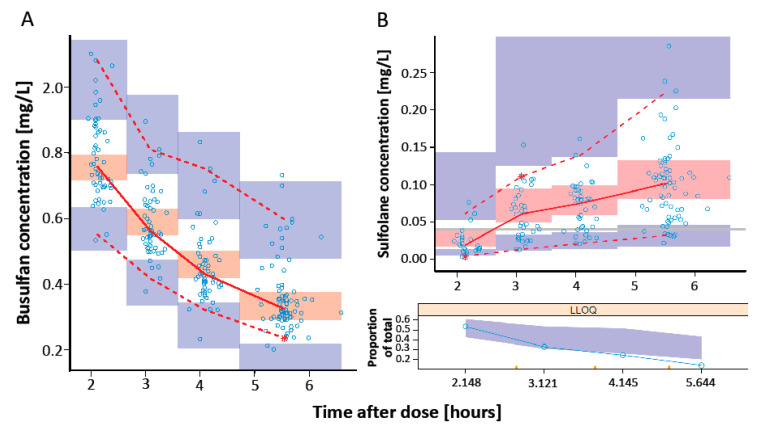
Prediction-corrected visual predictive check of the final model of busulfan (**A**) and sulfolane (**B**) with stratification on **Q6H**. Open dots represent prediction-corrected observed plasma concentration; solid red line = median observed concentration over time; dashed red lines = 5 and 95% quartiles of observed concentration over time; the * represent outliers at that timepoint; blue shaded area = 90% CI of 5% and 95% predictions; red shaded area = 90% CI of median predictions; horizontal grey line in (**B**) = the LLOQ of sulfolane (0.04 mg/L); lower panel in (**B**) shows the fraction of BQL sulfolane data.

SIR was performed with a M/m ratio of 5000/1000 and revealed adequate diagnostic plots with a proposal distribution close to the true distribution (Figure S2) and a horizontal trend for the observed resampling proportion (Figure S4). The pcVPC with stratification on Q6H showed overlapping observations and predictions for both busulfan (Figure 3A) and sulfolane (Figure 3B). The pcVPC plot with stratification on Q24H is provided in the Supplementary Materials (Figure S5).

Overall, the plots indicate a good predictive performance and robustness of the final model.

## 4. Discussion

This is the first study to describe population pharmacokinetics of busulfan in patients with myelofibrosis undergoing allo-HSCT. Moreover, this is the first study to incorporate sulfolane into a PopPK model of busulfan in order to establish a relationship between its metabolite and patient-specific covariates.

Our data suggests that the pharmacokinetics of busulfan and its metabolite sulfolane are best described by a one-compartment model with first-order elimination. This lies in accordance with most of the published PK analyses of busulfan, even though there are few two-compartmental models for busulfan reported as well [13,26,39,40]. Most published PopPK models of busulfan set the focus either on pediatric patients as the IIV as well as the IOV of busulfan PK in children and young adults are even more difficult to predict [20,21] or on large study populations including various malignancies [11,13]. However, there is no PopPK analysis solely focused on patients with myelofibrosis to date. Considering that patients with myelofibrosis have an elevated risk of hepatotoxicity and impaired liver function due to extramedullary hematopoiesis on the one hand, and the fact that an impaired liver function is associated with adverse impact on survival on the other hand [41], determining the inter-individual pharmacokinetic variability of busulfan in patients with myelofibrosis was overdue. The range of cAUC of busulfan (25.67–61.85 mg × h/L) in our study shows a up to 2.4-fold difference in busulfan exposure and, therefore, confirms the high IIV in drug exposure that is known from the literature as well. Additionally, even though busulfan/fludarabine, as either RIC or MAC, are commonly used conditioning regimens, there is still no defined therapeutic window for myelofibrosis.

There are only a few PopPK models of busulfan that solely include adult patients. As McCune et al. showed, there is a maturation of clearance in pediatric patients [13] and, consequently, the reported range for typical values of CL in the literature is considerably wide. Our results for CL_Bu_ (16.3 L/h) and V_Bu_ (61.5 L) for a typical patient with 75 kg TBW are generally within the range of the estimates reported in the literature. However, they differ from those of Choi et al., who found a CL of 11 L/h and V of 42.4 L for their adult patients (typical patient weighing 60 kg) [23]. Since Choi et al. included various malignancies in their analysis, the difference in population estimates might be an indicator of the necessity for more focused PK analyses on special patient populations. 

Regarding patient-specific variables, body size–related covariates and GSTA1 are, similar to our findings, most often reported to have a significant impact on the PK of busulfan. However, our study is the first to incorporate sulfolane into a PopPK model of busulfan. Although our findings did not confirm that a metabolic ratio of busulfan/sulfolane ≥5 is associated with a higher rate of graft failure and decreased event-free survival (EFS) [28], the fact that our data indicates an IIV on CL_Su_ of 112.8% CV underlines the complexity of busulfan’s metabolic pathway and calls for further investigations regarding the impact of metabolites on patient outcome as well. Moreover, the established relationship between GSTA1 and the clearance of the metabolite in our model may seem counterintuitive at first since sulfolane is not conjugated with glutathione. However, there are several intermediate metabolites within the pathway that are transitioned by different enzymes, and a change in any of the respective enzymes’ activity could potentially impact the excretion of sulfolane [21].

There are a few limitations to this study that need to be kept in mind. First, the rather small cohort of 37 patients might not allow us to adequately characterize the relationships between covariates and PK parameters, in particular if the covariate effects on PK parameters are of a small effect size. In addition, using a PopPK model based on a relatively small patient cohort for model-informed precision dosing (MIPD) is not advisable since the quantitative relationship between a covariate and its respective PK parameter might be imprecise and, therefore, lead to biased estimations of drug exposure [42]. Second, due to the nature of including seven patients retrospectively, we could not obtain sulfolane plasma concentrations for those patients in order to include them in the model. Third, for technical reasons, we were unable to conduct a Fibroscan in 14 patients, and therefore, a covariate relationship could not be sufficiently investigated.

## 5. Conclusions

To the best of our knowledge, this is the first PopPK model developed to describe busulfan’s pharmacokinetics in patients with myelofibrosis. TBW was identified as the most significant covariate. Incorporating its metabolite sulfolane into the model not only allowed us to characterize the covariate relationship between GSTA1 and the clearance of the metabolite but it also showed that there is a high inter-individual variability regarding CL_Su_ as well. Further (multi-centric) studies with larger cohorts are required in order to find further covariates that explain the high IIV of sulfolane CL and possibly determine a sensible therapeutic window for patients with myelofibrosis.

## Figures and Tables

**Table 1:** Patient demographic and clinical data.

Patient Characteristics (*n* = 37)	Median [IQR] or *n* (%)
Age [years]	60 [53.5–65.5]
Sex [female/male]	19 (51.4)/18 (48.6)
Weight [kg]	75 [64.05–88.25]
Height [cm]	174 [168–181]
BSA [m^2^]	1.84 [1.75–2.07]
*Diagnosis*	
PMF	18 (48.7)
Post-ET MF	9 (24.3)
Post-PV MF	10 (27)
*Dosing regime*	
Q6H	30 (81)
Q24H	7 (19)
*DIPSS/MYSEC*	
Intermediate-1	2 (5)/1 (3)
Intermediate-2	15 (40)/14 (38)
High Risk	1 (3)/4 (11)
*Mutation*	
JAK2	26 (70.3)
CALR	7 (18.9)
MPL	1 (3)
TET2	9 (24.3)
ASXL1	13 (35.1)
*Blood chemistry, serum levels*	
AST [U/L]	21 [15.5–31.5]
ALT [U/L]	21 [18.5–47.5]
De Ritis Ratio	0.76 [0.58–1.07]
Albumin [g/L]	37.8 [34.6–41.4]
Alkaline Phosphatase [U/L]	85 [63–115]
Bilirubin [mg/dL]	0.6 [0.5–0.8]
Fibroscan [kPa]	5.6 [4.8–7]
Missing Data	14 (37.8)
GSTA1 52G > A	28 (75.7)
GSTM1 Deletion	19 (51.35)
Mucositis Grade 1/2/3/4	10 (27)/14 (38)/1 (2.7)/1 (2.7)
aGvHD Grade 1/2/3	10 (27)/7 (19)/4 (11)
cGvHD Grade 1/2/3	12 (32)/6 (16)/1 (2.7)
SOS	2 (5.4)
Relapse	2 (5.4)
Death	5 (13.5)

**aGvHD**: acute graft-versus-host-disease; **ALT**: alanine transaminase; **AST**: aspartate transaminase; **cGvHD**: chronic graft-versus-host disease; **DIPSS**: Dynamic International Prognostic Scoring System; **MYSEC**: myelofibrosis secondary to PV and ET; **PMF**: primary myelofibrosis; **Post-ET MF**: post-essential thrombocythemia myelofibrosis; **Post-PV MF**: post-polycythemia vera myelofibrosis; **SOS**: sinusoidal obstructive syndrome.

**Table 2:** Parameter estimates of the final busulfan and sulfolane PK model and SIR results.

Parameters	Final Model			SIR (M/m = 5000/1000)
	Estimate	RSE (%)	Shrinkage (%)	95% CI
**CL_Bu_** [L/h]	16.3	3.6	-	15.18–17.35
**V_Bu_** [L]	61.5	2	-	59.37–63.78
**CL_Su_** [L/h]	1.61	37	-	0.84–2.24
**V_Su_** [L]	48.8	35.2	-	30.75–78.46
**MF**	0.0704	28.6	-	0.0463–0.1029
**COV_V_Bu__TBW** [kg]	0.854	11.6	-	0.665–1.059
**COV_CL_Su__GSTA1**	1.43	43.6	-	0.63–2.40
**IIV CL_Bu_** [CV%]	21.5	14.8	2	16.4–27.4
**IIV V_Bu_** [CV%]	10	12	18	7.2–12.1
**IIV CL_Su_** [CV%]	112.8	26.1	22	80.3–206.2
**IIV V_Su_** [CV%]	77.6	14.2	18	59.2–106.4
**IOV CL_Bu_** [CV%]	7.6	13.5	39	5.6–9.1
**Prop. σ_Bu_** [CV%]	7.1	12.8	14	6.3–8.2
**Prop. σ_Su_** [CV%]	36.2	7.2	12	32.6–40.1

**CL_Bu_**: busulfan clearance; **CL_Su_**: sulfolane clearance; **COV_CL_Su__GSTA1**: typical pharmacokinetic parameter for the covariate GSTA1 on CL_Su_; **COV_V_Bu__TBW**: typical pharmacokinetic parameter for the covariate BTW on V_Bu_; **CV**: coefficient of variation (%CV = sqrt(exp(OMEGA)-1) * 100); **IIV**: inter-individual variability; **IOV**: inter-occasion variability; **MF**: metabolic fraction; **OFV**: objective function value; **Prop. σ_Bu_**: residual variability of busulfan calculated as a proportional error; **Prop. σ_Su_**: residual variability of sulfolane calculated as a proportional error; **RSE**: relative standard error; **SIR**: sampling-importance resampling; **V_Bu_**: volume of distribution of busulfan; **V_Su_**: volume of distribution of sulfolane.

## Data Availability

The data presented in this study are available on request from the corresponding author. The data are not publicly available for reasons of privacy.

## Supplementary Materials

The following supporting information can be downloaded at: https://www.mdpi.com/article/10.3390/pharmaceutics14061145/s1, Figure S1: Individual busulfan clearance after the first and ninth dose; Figure S2: SIR diagnostic plot; Figure S3: SIR diagnostic plot: Adequacy of proposal density; Figure S4: SIR diagnostic plot: Exhaustion of samples; Figure S5: pcVPC with stratification on Q24H.
