# Supplementary material for: Population Pharmacokinetics of Busulfan and Its Metabolite Sulfolane in Patients with Myelofibrosis Undergoing Hematopoietic Stem Cell Transplantation
Source: Pharmaceutics. 2022 May 27;14(6):1145. doi: 10.3390/pharmaceutics14061145 (PMC9229330; doi:10.3390/pharmaceutics14061145)
Supplement: Supplementary file 1 [file pharmaceutics-14-01145-s001.zip › pharmaceutics-1724333-supplementary.pdf]

## Supplemental Material

### Population pharmacokinetics of Busulfan and its metabolite Sulfolane in patients with Myelofibrosis undergoing hematopoietic stem cell transplantation

Adrin Dadkhah[1], Sebastian Georg Wicha[2], Nicolaus Kröger[3], Alexander Müller[4], Christoph Pfaffendorf[2], Maria Riedner[5], Anita Badbaran[3], Boris Fehse[3], Claudia Langebrake[1,3]

[1]University Medical Center Hamburg-Eppendorf, Hospital Pharmacy, Hamburg, Germany,

[2]University of Hamburg, Dept. of Clinical Pharmacy, Institute of Pharmacy, Hamburg, Germany,

[3]University Medical Center Hamburg-Eppendorf, Department of Stem Cell Transplantation, Hamburg, Germany,

[4]University Medical Center Hamburg-Eppendorf, Department of Legal Medicine, Hamburg, Germany,

[5]University of Hamburg, Technology Platform Mass Spectrometry, Hamburg, Germany

### Bioanalytical method for quantification of sulfolane

The quantification of sulfolane was derived from the bioanalytical method of McCune et al. using a QTRAP 5500 mass spectrometer (SCIEX, Framingham, Massachusetts, USA) coupled with a 1290 Infinity HPLC II (Agilent Technologies, California, USA) [1].

A calibration from 0.04 to 1 mg/L using six calibrators was prepared for each measurement. An independently prepared quality control sample of sulfolane (0.2 mg/L) was analyzed in each run. The within-run and between-run inaccuracy and imprecision of the assay across all runs was < 9.6 % and < 9 %, respectively.

Solvents were used in LC-MS grade. Calibration standards and quality control samples were solved in methanol, diluted in water and spiked with plasma. All samples (100 µL) were processed for protein precipitation by adding 100 µL of cold 4% trichloroacetic acid in water and subsequently centrifuged for 10 minutes at 14,000 rpm at 4°C. Approximately 80 µL of the supernatant was transferred to the appropriate well of a 96 well microtiter plate and 5 µL were injected. Separation was performed on a Ascentis® Express 90 Å AQ-C18 column (150x3 mm, 5 µm particle size, Supelco Inc, Bellafonte, Pennsylvania, USA). Column temperature was set to 35°C. Solvents (A) ammonium formate 10 mM in water containing formic acid for adjusting to pH 3.5 and (B) methanol were used in a gradient program. The gradient conditions were as follows: starting conditions 5% B for 4 minutes, linear gradient starting at 5% B to 70% B within 2 min then isocratic at 70% B for 1 min, then linear gradient to 5% B over 1 min and then isocratic at 5% B for 2 min. Total run time was 10 min at a flow rate of 0.5 mL/min. The retention time of sulfolane was 2.6 min.

LC–MS/MS data were acquired and analyzed using Analyst 1.7 software (SCIEX, Framingham, Massachusetts, USA). The multiple reaction monitoring (MRM) transitions used were m/z 129.1/65.0 (target) and 121.1/47.8 (qualifier).

**Primer sets used for the genotyping assays**

**GSTA1 -52G>A (rs3957356):**

Forward primer: 5'-ACTGAATTCCAGGTCCTAATGTATTTATAAGC-3'
Reverse primer: 5'-GGCTTTTCCCTAACTTGACTCTTCT-3'
Probe primer: 5'-FAM-AGTGGGAGAGAACTA-MGBEQ-3'

**GSTM1 deletion:**

Forward primer: 5'-CTGAGCCCTGCTCGGTTTAG-3'
Reverse primer: 5'-ATGGGCATGGTGCTGGTT-3'
Probe: 5'-FAM-CTGTCTGCGGAATC-BHQ1-3'

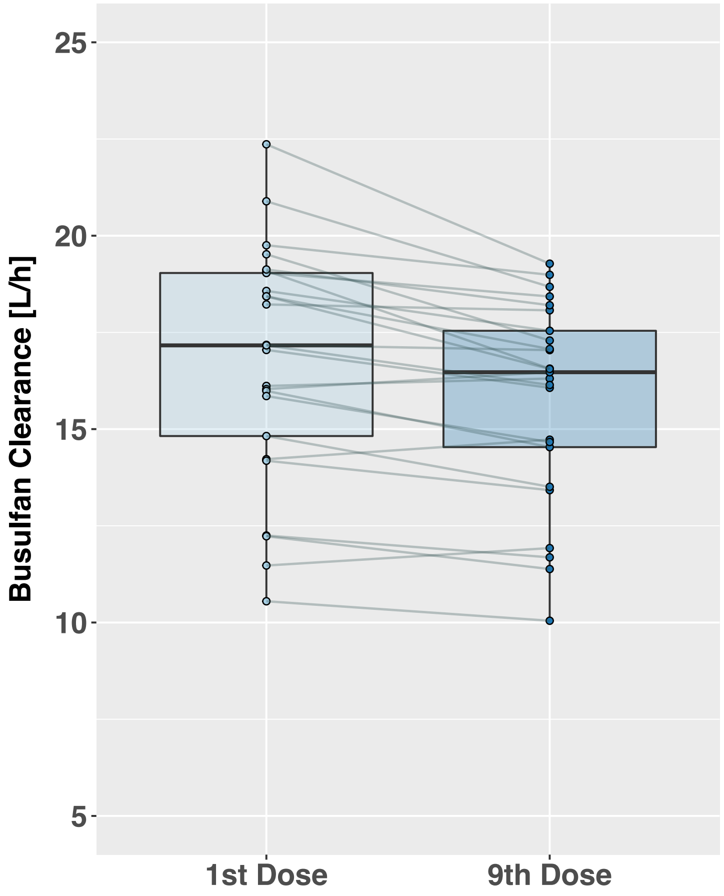


**Figure S1.** Individual busulfan clearance after the first and ninth dose

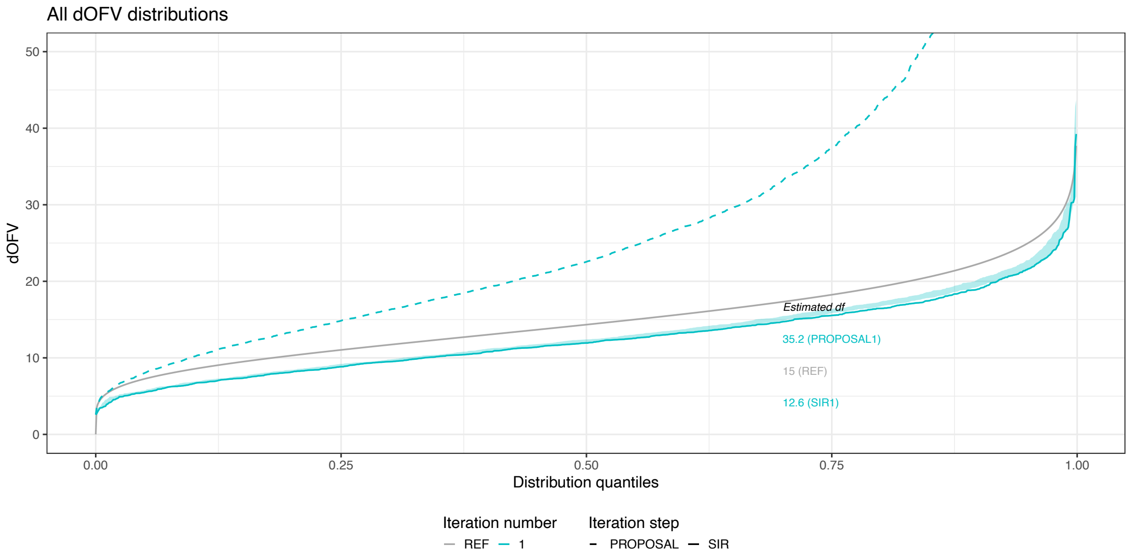


**Figure S2.** SIR diagnostic plot

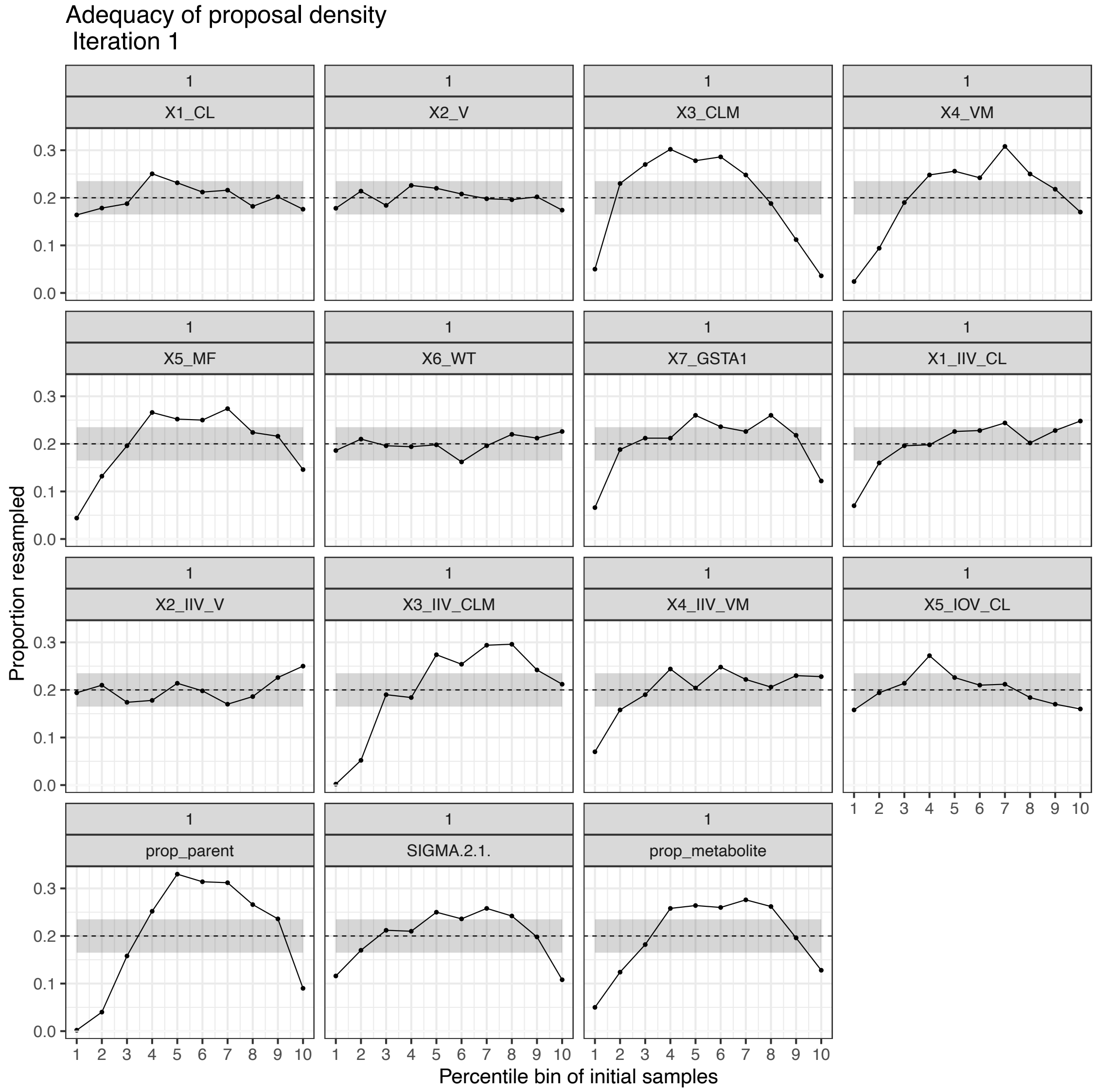


**Figure S3.** SIR diagnostic plot: Adequacy of proposal density

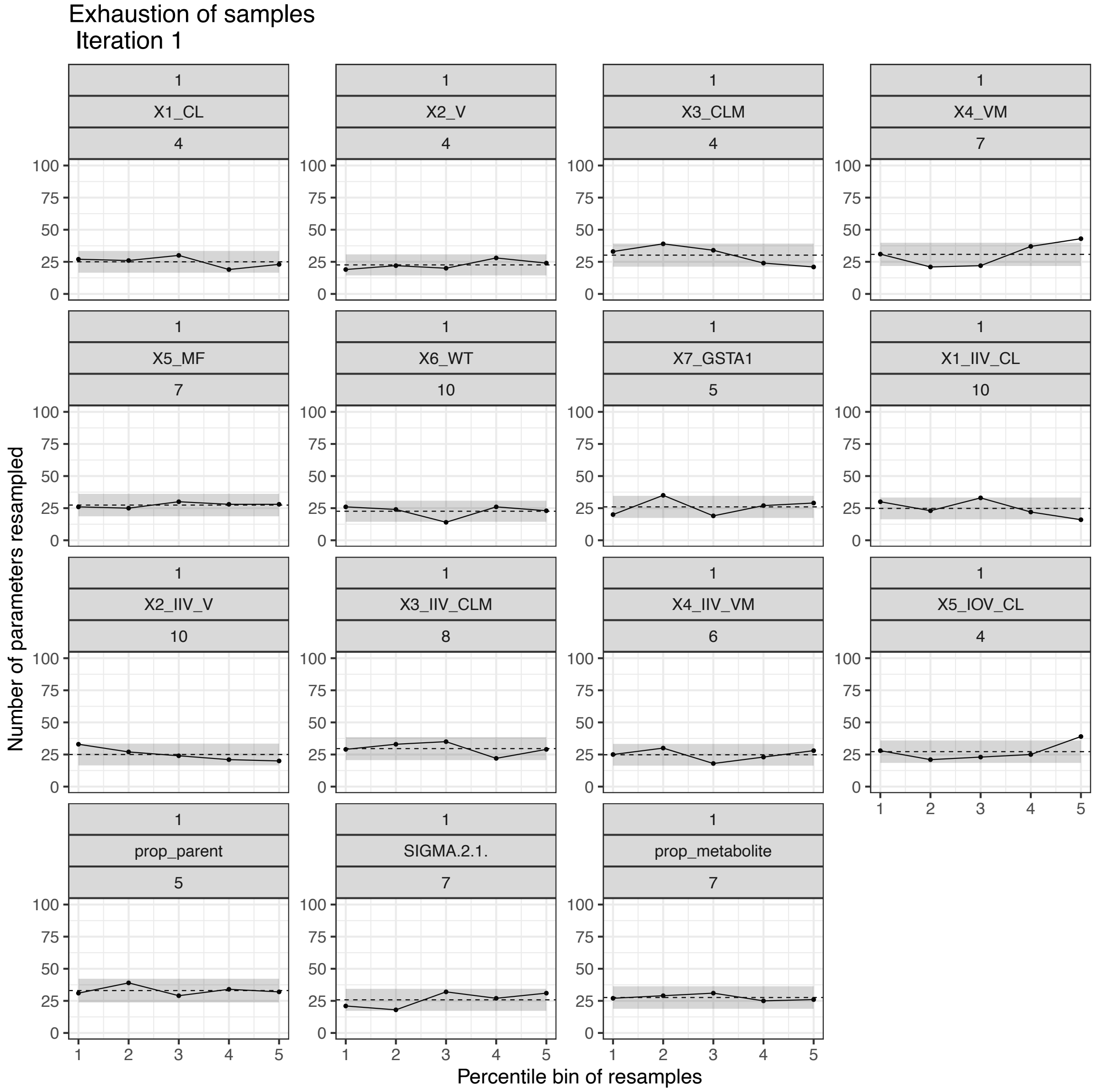


**Figure S4.** SIR diagnostic plot: Exhaustion of samples

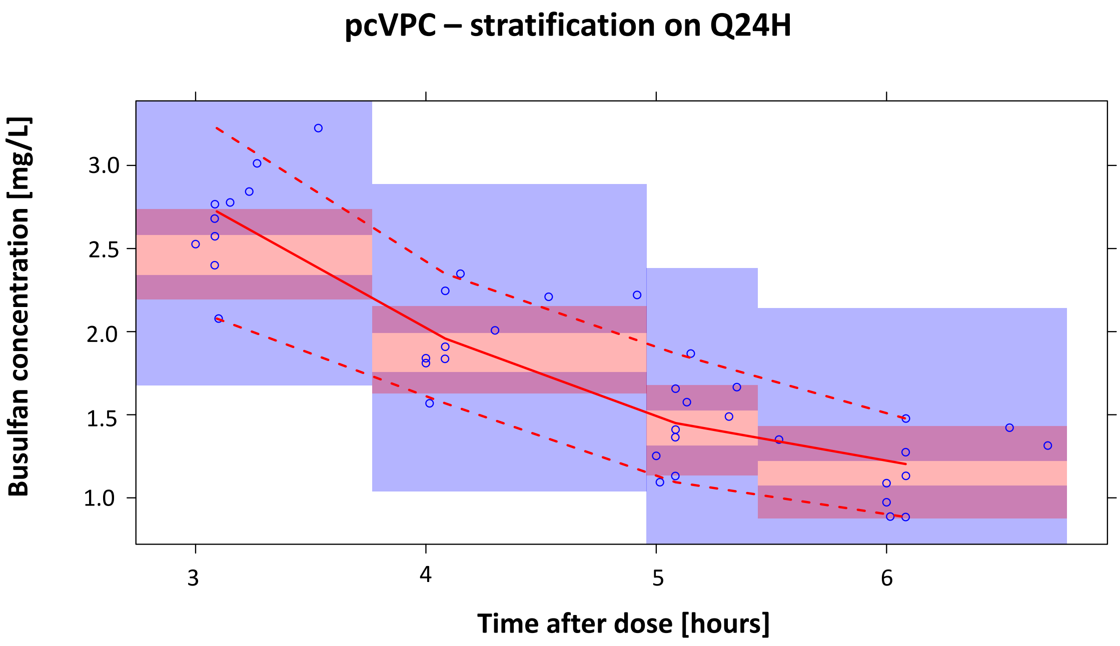


**Figure S5**. pcVPC with stratification on Q24H: Open dots represent prediction-corrected observed plasma concentration; solid red line: median observed concentration over time; dashed red lines: 5 and 95 % quartiles of observed concentration over time; blue shaded area: 90 % CI of 5 % and 95 % predictions; red shaded area: 90 % CI of median predictions

1. McCune J, Shen DD, Shireman L, Phillips B. Bioanalytical Method: Tetrahydrothiophene-1-oxide and Sulfolane and 3-Hydroxysulfolane in Plasma. University of Washington School of Pharmacy; Report No.: BAM217; National Cancer Institute [R01 CA182963] (to J.S.M.).
